# Chatting online – is this good for you?

**DOI:** 10.1186/1471-2334-13-S1-O4

**Published:** 2013-12-16

**Authors:** Ana Maria Tudor, Mariana Mărdărescu, Ruxandra Neagu-Drăghicenoiu, Rodica Ungurianu, Adrian Abagiu, Carina Matei, Ioana-Catrinel Cercel, Cristian Anghelina, Mioara Predescu

**Affiliations:** 1 National Institute for Infectious Diseases “Prof. Dr. Matei Balş”, Bucharest, Romania; 2 Carol Davila University of Medicine and Pharmacy, Bucharest, Romania; 3 Romanian HIV/AIDS Center, Bucharest, Romania

## Background

We aim to describe physicians’ experience as a part of web conferences project involving health care practitioners, governmental and nongovernmental organizations targeting health education among people living with HIV (PLHIV).

## Methods

We analyze different aspects of a project developed by nongovernmental organizations for people affected by HIV, namely the Romanian Anti-AIDS Association (ARAS), the National Union of Organizations of People Affected by HIV/AIDS (UNOPA) along with the Romanian HIV/AIDS Center and the National Institute for Infectious Diseases “Prof. Dr. Matei Balş”. Medical, psychological and social support was given by Matei Balş personnel. The conferences offered direct connection between AIDS care specialists and patients. The IT logistics was provided by Data Center Solutions.

## Results

Two series of conferences consisting of six sessions provided information for more than 1000 visitors. In each session there were 2 health care practitioners, in total 5 physicians, 2 psychologists and one social worker. The main themes proposed by AIDS experts were: HIV transmission, treatment, adherence, parenting for HIV infected children, emotional problems faced by HIV patients, social and psychological support, IV drug users, antiretrovirals’ adverse reactions, pregnancy and HIV, hepatitis C coinfection. Patients’ focus was on metabolic problems associated with antiretroviral treatment, legal rights for HIV infected persons, HIV cure, antiretroviral supplies, protection for partners and future children.

## Conclusion

Health care practitioners had the opportunity to identify the problems faced by patients, their concerns. Patients took advantage from asking questions under protection of undisclosed identity. The feedback from both parties, patients and AIDS experts was positive. All the partners involved benefit from this project.

